# MutS homologue hMSH4: interaction with eIF3f and a role in NHEJ-mediated DSB repair

**DOI:** 10.1186/1476-4598-12-51

**Published:** 2013-06-02

**Authors:** Yen-Lin Chu, Xiling Wu, Yang Xu, Chengtao Her

**Affiliations:** 1 School of Molecular Biosciences, College of Veterinary Medicine, Washington State University, Mail Drop 64–7520, Pullman, WA 99164, USA

**Keywords:** hMSH4, eIF3f, Ionizing radiation (IR), Non-homologous end-joining (NHEJ)

## Abstract

**Background:**

DNA mismatch repair proteins participate in diverse cellular functions including DNA damage response and repair. As a member of this protein family, the molecular mechanisms of hMSH4 in mitotic cells are poorly defined. It is known that hMSH4 is promiscuous, and among various interactions the hMSH4-hMSH5 interaction is involved in recognizing DNA intermediate structures arising from homologous recombination (HR).

**Results:**

We identified a new hMSH4 interacting protein eIF3f – a protein that functions not only in translation but also in the regulation of apoptosis and tumorigenesis in humans. Our studies have demonstrated that hMSH4-eIF3f interaction is mediated through the N-terminal regions of both proteins. The interaction with eIF3f fosters hMSH4 protein stabilization, which in turn sustains γ-H2AX foci and compromises cell survival in response to ionizing radiation (IR)-induced DNA damage. These effects can be, at least partially, attributed to the down-regulation of NHEJ activity by hMSH4. Furthermore, the interplay between hMSH4 and eIF3f inhibits IR-induced AKT activation, and hMSH4 promotes eIF3f-mediated bypass of S phase arrest, and ultimately enhancing an early G2/M arrest in response to IR treatment.

**Conclusion:**

Our current study has revealed a role for hMSH4 in the maintenance of genomic stability by suppressing NHEJ-mediated DSB repair.

## Background

Successful and timely repair of DNA DSBs is critically important for the maintenance of genomic stability and cell survival. Defective DSB repair, often as a consequence of mutations in DSB repair genes, is closely associated with genomic and chromosomal abnormalities and is a high risk factor for cancer development [1-3]. The molecular mechanisms that are involved with DSB repair have become increasingly complex – in particular this process can be regulated at various levels by many different protein factors [4]. The regulation, in most of cases, is achieved through protein interactions, subcellular localizations, and post-translational modifications.

It is evident that mammalian cells could resolve DSBs in a number of ways, in which HR and NHEJ represent the two predominant DSB repair pathways [1,2]. Generally, the HR pathway carries out error-free DSB repair in either S or G2 phases of the cell cycle, in which a homologous repair template (*i*.*e*. donor sequence) on a homologous chromosome or a sister chromatid is utilized to restore the integrity of the broken chromosome [1]. It is presently conceived that HR-mediated DSB repair can be accomplished by at least two main mechanisms – the classic double-strand break repair (DSBR or double-Holliday junction) and the synthesis-dependent strand annealing (SDSA) pathways. DSBR will give rise to unique sequence configurations known as crossover and non-crossover, whereas SDSA-mediated DSB repair will not be expected to alter HR donor sequences. The HR pathway is purportedly critical for the repair of one-ended DSBs that may arise when replication forks encounter single-strand breaks [4], in which the error-prone NHEJ is presumably less favorable. In addition, DSB repair mediated by the error-prone NHEJ pathway is frequently associated with deletions at the repair joints due to the processing of DNA ends before rejoining [2]. It is commonly accepted that NHEJ can occur throughout all cell cycle phases; however, very little is known about how NHEJ or HR is selected at S and G2 phases when both pathways are operational.

Besides functioning in DNA mismatch repair (MMR), members of MMR family also play important roles in modulating DSB repair [5-10]. Evidently, the MutS homologue proteins hMSH2 and hMSH6 (together with hMLH1) coexist with proteins involved in DSB repair in a mega protein complex [11], and the involvement of these MMR proteins in the process of DSB repair has been suggested by many studies [5-10]. Interestingly, two of the MutS homologue proteins, hMSH4 and hMSH5, do not appear to function in MMR but rather participate in the process of HR [12-16], and a role of hMSH5 in mitochondria DNA repair has been recently suggested [17]. Although Msh4 and Msh5 null mutations share similar meiotic HR defects in mice, evidence suggests that hMSH4 and hMSH5 can exert distinct functions in mitotic cells [12]. In particular, hMSH4 interacts with an array of proteins—including hMSH5, VBP1, hMLH1, hMLH3, hRad51, DMC1 and GPS2—known to function in several aspects of the DNA damage response [13,15,18-23]. However, the expression levels of hMSH4 vary dramatically in different cell types – with a moderate expression in the testis and relatively low levels in other tissues including ovary, thymus, colon, pancreas, brain, liver, and placenta [20,24]. These observations raised the possibility that, by interacting with different binding partners or through varying the levels of protein expression, hMSH4 may exert diverse cellular functions. For example, hMSH5-binding stabilizes hMSH4 in the nucleus, whereas VBP1 competes with hMSH5 to bind hMSH4 and assists in its localization to the cytoplasm [20,25]. Here, we demonstrate that hMSH4 interacts with eIF3f – a regulatory subunit of the eIF3 complex that has also been implicated in the regulation of apoptosis and tumorigenesis in humans [26-32]. This interaction stabilizes hMSH4 in both the cytoplasm and the nucleus. Our study indicates that, through interacting with eIF3f, hMSH4 inhibits NHEJ-mediated DSB repair, therefore sensitizing cells to IR-induced DNA damage.

## Results

To explore the functional roles of hMSH4, we have identified eIF3f as a new hMSH4 interacting partner. Until recently, eIF3f was known as a conserved factor among *Caenorhabditis elegans*, *Drosophila melanogaster*, *Arabidopsis thaliana*, and *Homo sapiens*, suggesting its involvement in the “core” process of translation initiation [33,34]. Here we demonstrate that eIF3f stabilizes the hMSH4 protein in cells, thereby allowing hMSH4 to act in the process of DNA damage response and repair.

### hMSH4 interacts with eIF3f

**Figure 1:**
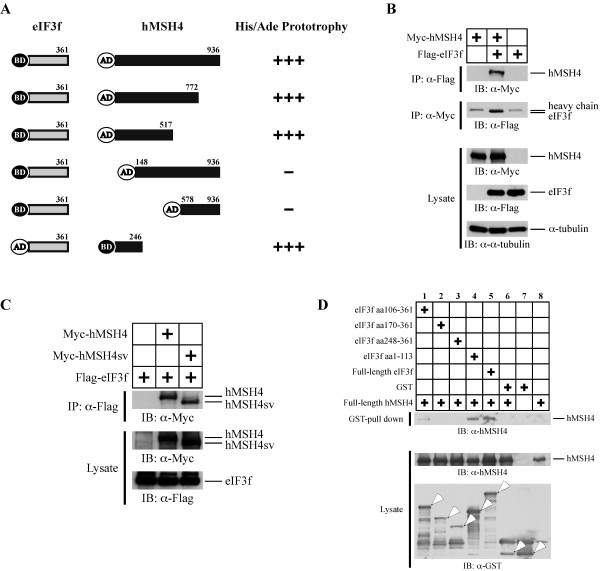
**The interaction between hMSH4 and eIF3f.** (**A**) Yeast-two hybrid analysis of the hMSH4-eIF3f interaction. A series of hMSH4 truncation mutants were utilized to determine the eIF3f-interacting domain on hMSH4. Positive interactions were ascertained by the transcription activation of *ADE2* and *HIS3* reporter genes. (**B**) Co-IP analysis of hMSH4-eIF3f interaction in human cells. Immunoblotting with α-tubulin was used as a loading control. (**C**) Co-IP analysis of the interaction between eIF3f and hMSH4sv. Full-length hMSH4 and hMSH4sv were expressed separately with eIF3f and the respective interactions were analyzed by co-IP. (**D**) GST pull-down analysis of hMSH4-eIF3f interaction and determination of the hMSH4-interacting domain on eIF3f. The full-length eIF3f and four truncated fragments were expressed as GST-fusion proteins. GST pull-down experiments were performed with Glutathione-Sepharose 4B beads.

To identify potential hMSH4 interacting proteins, we performed a yeast two-hybrid screening of the human ovary cDNA library using the full-length hMSH4 as bait [20]. This approach identified four individual clones containing eIF3f ORF sequences. One of the full-length clones was used for all subsequent studies. As shown in Figure 1A, yeast two-hybrid analysis demonstrated that hMSH4 specifically interacted with eIF3f. To determine the region of hMSH4 that could mediate the interaction with eIF3f, further yeast two-hybrid analysis was performed with a series of truncated hMSH4 fragments. The results presented in Figure 1A clearly indicated that the interaction between hMSH4 and eIF3f was mediated through the N-terminal region of hMSH4 – most likely involving the first 150 amino acids of hMSH4. Since the known hMSH4 domains involved in heterotypic and homotypic interactions are not located in the N-terminal region of hMSH4 [20,22,35], the binding of eIF3f to hMSH4 is unlikely to affect the hMSH4-hMSH5 or the homotypic hMSH4 interactions.

To validate the hMSH4-eIF3f interaction in human cells, we co-expressed Myc-tagged hMSH4 and Flag-tagged eIF3f in 293T cells (Figure 1B). Co-immunoprecipitation (co-IP) with either the anti-Myc or the anti-Flag antibody was performed, and the results of co-IP analysis demonstrate that hMSH4 interacts with eIF3f in human cells (Figure 1B). Furthermore, consistent with the results of the yeast two-hybrid analysis, similar co-IP experiments in 293T cells show that the C-terminal region of hMSH4 is not involved in the eIF3f interaction. Specifically, Myc-hMSH4sv, an alternative splicing variant lacking the C-terminal end [20], was equally competent to interact with Flag-eIF3f as that of the full-length hMSH4 (Figure 1C).

To further validate the existence of a direct physical interaction between hMSH4 and eIF3f, we next performed a GST pull-down analysis of recombinant His_6_-hMSH4 and GST-eIF3f proteins. The results of this assay confirmed that hMSH4 directly interacts with eIF3f (Figure 1D, lane 5). To determine which region of eIF3f is responsible for hMSH4 interaction, a series of truncated eIF3f mutants were tested. The results showed that the N-terminal region of eIF3f (*i*.*e*., aa1-113) possesses an equivalent hMSH4 binding capacity to that of the full-length eIF3f, while the overlapping fragment aa106-361 displayed a low binding activity (Figure 1D). Together, these results indicate that hMSH4 interacts with eIF3f, and the N-terminal regions of both proteins are involved in this interaction.

### eIF3f facilitates hMSH4 stabilization

**Figure 2:**
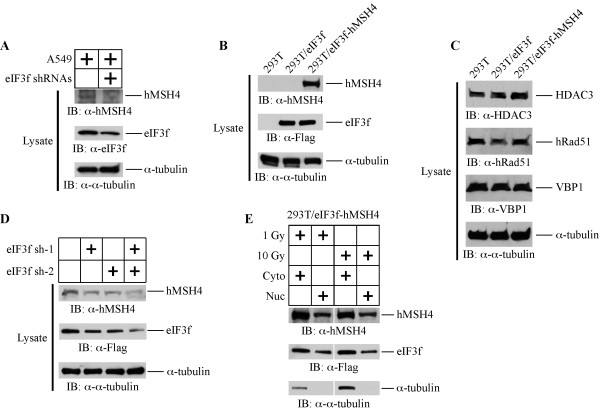
**eIF3f facilitates hMSH4 stabilization.** (**A**) The effect of RNAi-mediated down-regulation of eIF3f on the levels of endogenous hMSH4 was analyzed in A549 cells. A mixture of eIF3f sh-1 and sh-2 RNAi constructs was used for transient transfection, and cells were collected and analyzed by immunoblotting at 48 hrs post-transfection. Immunoblotting with α-tubulin was used as a loading control. (**B**) Stable expression of eIF3f and hMSH4 in 293T/eIF3f and 293T/eIF3f-hMSH4 cell lines (from selected single clones). (**C**) Western blotting analysis of the levels of HDAC3, hRad51, and VBP1 expression in 293T, 293T/eIF3f, and 293T/eIF3f-hMSH4 cells. (**D**) Effects of reduced eIF3f expression on the levels of hMSH4 in the stable cell line 293T/eIF3f-hMSH4. Reduction of eIF3f expression was achieved by transient transfection of eIF3f RNAi constructs. (**E**) Nuclear and cytoplasmic distribution of hMSH4 and eIF3f proteins in response to IR. 293T/eIF3f-hMSH4 cells treated with 1 or 10 Gy IR were fractionated at 6 hrs post-treatment and the levels of hMSH4 and eIF3f in the nuclear and cytoplasmic fractions were determined by immunoblotting. α-tubulin was used as a marker for the cytoplasmic fraction.

To understand the function of the eIF3f-hMSH4 interaction, we examined whether eIF3f promoted the stabilization of hMSH4. Specifically, A549 cells were treated with a mixture of eIF3f RNAi knockdown constructs (eIF3f sh-1 and eIF3f sh-2) and the levels of hMSH4 protein were analyzed (Figure 2A). Interestingly, the endogenous hMSH4 level was reduced corresponding to a decrease in eIF3f, suggesting that eIF3f can potentially stabilize hMSH4 protein in cells (Figure 2A). However, the levels of hMSH4 expression in various cell lines are generally on the borderline of immuno-detection limit [12]. Thus, we have generated stable cell lines to further investigate the effects of the eIF3f-hMSH4 interaction on hMSH4 stabilization (Figure 2B). Although transient expression of hMSH4 in 293T cells was readily achievable, we failed at numerous attempts to create an hMSH4 stable cell line – drug resistant colonies were obtained but none expressed hMSH4 protein. On the contrary, we were able to generate a stable cell line expressing both eIF3f and hMSH4, *i.e.* 293T/eIF3f-hMSH4, suggesting a role for eIF3f in hMSH4 protein stabilization (Figure 2B). Clearly, eIF3f *per se* was not sufficient enough to upregulate the expression of hMSH4 in 293T/eIF3f cells (Figure 2B). Immunoblotting analysis of 293T, 293T/eIF3f, and 293T/eIF3f-hMSH4 cell lines indicated that eIF3f or hMSH4 overexpression did not affect the levels of HDAC3, hRad51, and VBP1 – proteins known to associate with hMSH4 (Figure 2C).

To further confirm that eIF3f could affect hMSH4 stability, the levels of eIF3f in 293T/eIF3f-hMSH4 cells were reduced by eIF3f RNAi, and the levels of hMSH4 were examined by immunoblotting. As shown in Figure 2D, the reduction of eIF3f protein was clearly correlated with a decrease in hMSH4. In particular, RNAi-mediated effective eIF3f reduction (via both eIF3f sh-1 and sh-2) led to a significant decrease in hMSH4 levels (Figure 2D). Evidently, both hMSH4 and eIF3f were present in the nuclear and cytoplasmic fractions, and this protein distribution pattern was not altered by IR treatments (Figure 2E). Taken together, although these experiments did not completely rule out a potential indirect effect of eIF3f on hMSH4 stabilization, the results suggest that hMSH4 and eIF3f co-exist in both the nucleus and cytoplasm, and eIF3f facilitates the stabilization of hMSH4 in cells.

### hMSH4 reduces cell survival and compromises DSB repair in response to IR treatment

**Figure 3:**
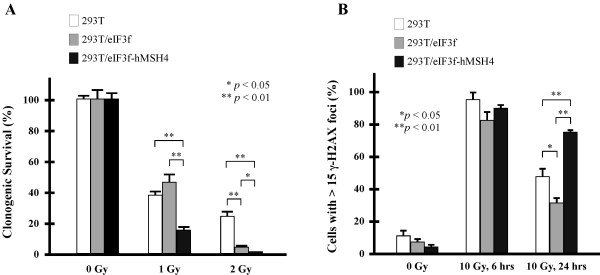
**Effects of eIF3f**-**hMSH4 on cellular response to IR.** (**A**) Clonogenic survival analysis of 293T, 293T/eIF3f and 293T/eIF3f-hMSH4 cells treated with 1 or 2 Gy IR. Colonies that contained at least 50 cells were counted and the percentage of cell survival was determined in reference to untreated control cells. The means of three individual experiments and the corresponding standard deviations (error bars) are presented. (**B**) Examination of γ-H2AX foci formation at 6 or 24 hrs post-exposure to 10 Gy IR. Percentages of cells possessing 15 or more foci/nucleus are graphically presented, and statistically significant differences are indicated with asterisks (*p < 0.05 and **p < 0.01; Student’s *t*-test).

To explore the potential role of hMSH4-eIF3f in cellular response to DNA damage, clonogenic survival and γ-H2AX foci analyses were performed with IR-treated cells. Clonogenic survival analysis indicated that eIF3f-hMSH4 significantly increased cellular sensitivity to IR treatments (Figure 3A). It is interesting to note that hMSH4 specifically sensitized cells to 1 Gy IR (Figure 3A), while eIF3f displayed no significant effect (Figure 3A). Conversely, eIF3f significantly increased the sensitivity of cells treated with 2 Gy IR and hMSH4 substantially promoted this effect (Figure 3A).

To investigate whether the altered survival response is related to compromised DSB repair in eIF3f-hMSH4 cells, we next analyzed the retention of IR-induced γ-H2AX foci – a surrogate indicator for compromised DSB repair [36]. We found that most cells (> 80%) of the 293T, 293T/eIF3f, and 293T/eIF3f-hMSH4 populations were γ-H2AX positive at 6 hrs following a treatment with 10 Gy IR (Figure 3B), suggesting similar DNA damage signaling in these cells. However, at 24 hrs post-IR, 293T/eIF3f-hMSH4 cells displayed the highest level of γ-H2AX foci retention while 293T/eIF3f cells possessed a lower level of γ-H2AX staining in comparison to that of 293T cells (Figure 3B). These observations indicate that hMSH4 (with the assistance of eIF3f) delays the repair of IR-induced DSBs, and thereby impeding cell survival in response to IR.

### hMSH4 exerts a strong inhibitory effect on NHEJ

**Figure 4:**
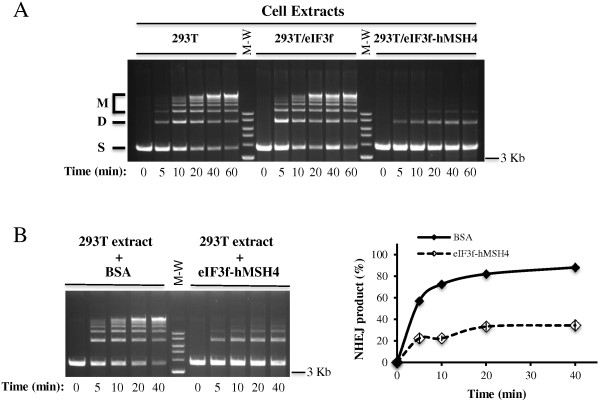
***In vitro *NHEJ assay.** (**A**) Determination of NHEJ activities in extracts prepared from 293T, 293T/eIF3f and 293T/eIF3f-hMSH4 cells. DNA end joining reactions were performed by incubation of cell extracts with *Sal*I-digested plasmid DNA, and these reactions were terminated at the indicated time points. End joining products were separated by agarose gel electrophoresis. ‘S’ signifies linear DNA substrate, ‘D’ for joint dimer, and ‘M’ indicates all other higher order joint products. (**B**) Analysis of NHEJ activities in 293T extracts complemented with either 5 μg of BSA or 293T/eIF3f-hMSH4 extracts under identical buffer conditions. A representative gel image was shown on the left, and the relative NHEJ activities were quantified and graphed as a function of time (on the right).

It is known that the repair of IR-induced DSBs is largely dependent on NHEJ in mammalian cells [2]. Therefore, to provide direct evidence implicating a role for hMSH4 in the regulation of IR-induced DSB repair, we performed *in vitro* NHEJ analysis with cell extracts prepared from these cell lines. As demonstrated in Figure 4A, extracts of 293T and 293T/eIF3f cells exhibited comparable time-dependent NHEJ activities toward a linearized DNA substrate – generating both end-joined dimers and multimers. Conversely, the NHEJ activity was much lower in 293T/eIF3f-hMSH4 cells (Figure 4A), suggesting that the presence of hMSH4 significantly repressed NHEJ-based DSB repair activity. To substantiate the above observation and most importantly to rule out potential variations that could be introduced during extract preparation, *in vitro* NHEJ assay was also performed with 293T extracts complemented with either BSA or 293T/eIF3f-hMSH4 extracts. The results of this set of experiments clearly demonstrated that, in comparison to BSA, addition of eIF3f-hMSH4 extracts caused a >50% reduction of the NHEJ activity in 293T extracts (Figure 4B).

**Figure 5:**
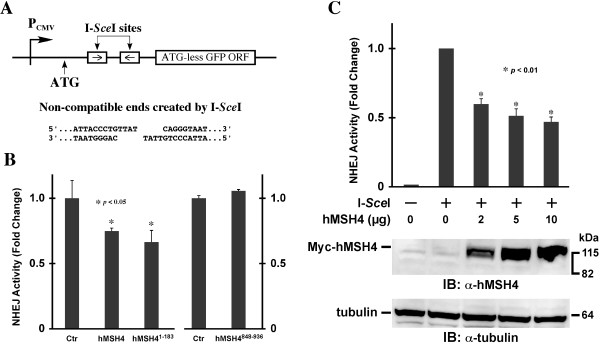
***In vivo *NHEJ analysis.** (**A**) Schematic illustration of the NHEJ reporter locus. The ATG start codon, located upstream of the I-*Sce*I recognition sites, is not in-frame with the GFP coding sequence. The relative location of the CMV promoter (P_CMV_) is indicated. (**B**) Analysis of the effect of hMSH4 on NHEJ. Plasmids encoding I-*Sce*I and the full-length hMSH4 or hMSH4 aa1-183 and aa848-936 fragments were co-transfected into the NHEJ reporter cell line 293T/#8-1. Transfected cells were analyzed by FACS at 48 hrs post-transfection. Average NHEJ activities of three independent experiments were graphed. Error bars are standard deviations from the means. (**C**) Dose-dependent effect of hMSH4 on NHEJ. Increased amounts of hMSH4 expression construct were transfected into NHEJ reporter cells at 24 hrs prior to I-*Sce*I transfection. FACS analysis was performed at 48 hrs post-I-*Sce*I transfection. Error bars represent standard deviations from the means of triplicate experiments. Western blotting analysis was performed to validate the increased levels of hMSH4 expression in the reporter cells.

To further authenticate the observed inhibitory effect of hMSH4 on NHEJ, we next utilized a NHEJ reporter cell line, 293T/#8-1, possessing a chromosomal NHEJ locus. As depicted in Figure 5A, the NHEJ locus harbors two inverted I-*Sce*I recognition sites that separate the start codon ATG and the linker region immediately connected to the ATG-less GFP coding sequence. Thus, NHEJ-mediated DSB repair at the I-*Sce*I cleavage sites can lead to the production of GFP expressing cells. As shown in Figure 5B, the results of the NHEJ reporter assay have indicated that transient expression of the full-length hMSH4 or the N-terminal hMSH4 fragment, aa1-183, can effectively suppress NHEJ; whereas the C-terminal hMSH4 fragment, aa848-936, has no significant effect. Since the N-terminal region of hMSH4 does not possess any hMSH5 binding activity [20,22], the regulatory effect of hMSH4 on NHEJ has to be hMSH5-independent. Consistent with this, the suppressive effect of hMSH4 on NHEJ is correlated with the amount of hMSH4 protein expression (Figure 5C). Together, these results indicate that hMSH4 possesses a direct suppressive effect on NHEJ-mediated DSB repair.

### hMSH4-eIF3f promotes an IR-induced G2/M arrest through suppressing AKT activation

**Figure 6:**
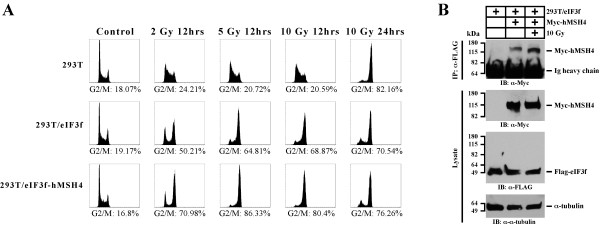
**Effects of eIF3f-hMSH4 on IR-induced cell cycle arrest.** (**A**) Cell cycle analysis of 293T, 293T/eIF3f, and 293T/eIF3f-hMSH4 cells treated with different doses of IR. Cell cycle analysis was conducted either at 12 hrs or 24 hrs post-IR treatment, and percentages of cells in the G2/M phase are indicated. (**B**) Effect of IR treatment on eIF3f-hMSH4 interaction. 293T/eIF3f cells were transfected to express Myc-hMSH4, and cells were then irradiated with 10 Gy IR at 48 hrs post-transfection. Cell lysates were prepared, 2 hrs post-IR treatment, for subsequent co-IP analysis.

In response to DNA damage, cells can activate several checkpoint mechanisms that are associated with different outcomes [37], of which one common consequence is cell cycle arrest. In light of the negative effects of hMSH4-eIF3f have on cell survival and NHEJ repair of IR-induced DSBs, we have next investigated their effects on cell cycle regulation in response to IR. As shown in Figure 6, IR-treated 293T cells first displayed S phase arrest at 12 hrs followed by a profound G2/M arrest at 24 hrs post-treatment, whereas 293T/eIF3f and 293T/eIF3f-hMSH4 cells displayed an early G2/M arrest. Specifically, 293T/eIF3f cells elicited a bypass of IR-induced S phase arrest and an early G2/M arrest, while hMSH4 expression promoted a more pronounced G2/M arrest in response to IR (Figure 6A). These results suggest that eIF3f-hMSH4 is also involved in the modulation of IR-induced DNA damage response, presumably facilitated by the enhanced hMSH4-eIF3f interaction following IR treatment (Figure 6B).

**Figure 7:**
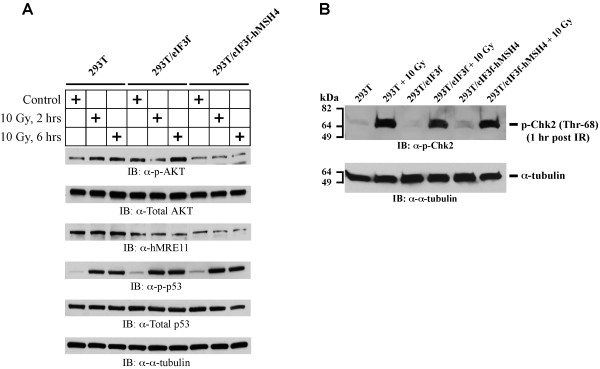
**Immunoblotting analysis of IR-induced AKT (Ser473) activation.** (**A**) The levels of AKT activation were measured by AKT Ser473 phosphorylation in 293T, 293T/eIF3f and 293T/eIF3f-hMSH4 cells treated with 10 Gy IR in comparison to untreated controls. Levels of p53 Ser15 phosphorylation and the total protein levels of AKT, hMRE11 and p53 were also analyzed. α-tubulin was used as a loading control. (**B**) The levels of Chk2 activation (Chk2 Thr68 phosphorylation) in 293T, 293T/eIF3f and 293T/eIF3f-hMSH4 cells in response to 10 Gy IR. Cell lysates were prepared at 1 hr post IR treatment. Untreated cells were analyzed as controls, while α-tubulin was used as a loading control.

Since IR-triggered AKT phosphorylation/activation exerts a negative effect on G2/M checkpoint activation [38,39], we next explored the possibility that eIF3f-hMSH4 might interfere with AKT-mediated DNA damage response. Specifically, the levels of IR-triggered AKT phosphorylation at Ser473 in these cell lines were evaluated. In comparison to 293T and 293T/eIF3f cells, although the levels of total AKT remain the same, IR treatment had little effect on AKT phosphorylation in 293T/eIF3f-hMSH4 cells (Figure 7A). These observations suggest that hMSH4 blocks DNA damage-induced AKT activation, thereby loosening the suppressive effect of AKT on G2 checkpoint activation – a possible underlying mechanism by which hMSH4 promotes G2/M arrest (Figure 6A). However, it is currently unclear how eIF3f mediates the bypass of IR-induced S phase arrest. Also of note are the recent observations connecting AKT phosphorylation with the levels of hMRE11 in both DSB repair and DNA damage response processes [40,41]. Consistent with these reports, we observed that the reduction of hMRE11 expression in 293T/eIF3f-hMSH4 cells correlated with the lack of IR-induced AKT phosphorylation (Figure 7A). Clearly, the levels of IR-induced p53 phosphorylation at Ser15 were very similar in the three cell lines (Figure 7A). In addition, in response to IR treatment, Chk2 phosphorylation at Thr68—an early DNA damage response event—was essentially identical in the three cell lines (Figure 7B). These results suggest that the unique effects of eIF3f-hMSH4 are not dependent on p53 and Chk2. Taken together, the data support that the interplay between hMSH4 and eIF3f specifically inhibits IR-induced AKT activation, and hMSH4 promotes an early G2/M arrest mediated by eIF3f in response to IR-induced DNA damage.

## Discussion

Understanding of DSB repair regulation in human cells is not only critical for revealing molecular events underlying genomic instability in cancer cells but also essential for devising more effective anti-cancer strategies. This is mainly because cancer is largely initiated and driven by somatic mutations and chromosomal rearrangements in affected cells. These genomic alterations can be largely attributed to excessive DNA damage, faulty DNA repair, and/or deregulated DNA repair activities. Interestingly, it has been recently proposed that, in addition to a gradual accumulation over time, the enormous genomic aberrations frequently occurred in cancer cells can also be generated by a one-off catastrophic event that dramatically alters genomic architecture [42]. Although little is known about the nature of this catastrophic event, it is believed that this has to be engaged with the formation and processing of dsDNA breaks by aberrant DSB repair.

Although loss-of-function mutations in DNA repair genes are the major etiologic factors for genomic instability in cancer cells, accumulation of high levels of genomic alteration within a short period of time at early stages of tumorigenesis would have to be facilitated by abnormally up-regulated error-prone DSB repair activities. In addition, unharnessed up-regulation of DNA repair activity can also increase the odds of developing antineoplastic resistance in cancer cells. However, our understanding of how cells normally control a proper level of DSB repair activity is presently very limited insofar as only a few studies have begun to address the potential mechanisms in controlling over-activation of DNA repair activities [8-10,43].

Here we have identified eIF3f as a novel binding partner for hMSH4. Even though eIF3f was originally characterized as a component of the translation initiation factor eIF3, recent studies have indicated that eIF3f may possess diverse cellular functions in proliferation and apoptosis [28,31,44]. In addition, studies have also indicated that eIF3f can regulate protein translation at various steps [29,32,45-47]. Increased levels of eIF3f expression are found to compromise proliferation and promote apoptosis in cancer cells [29], of which this pro-apoptotic property of eIF3f is presumably dependent on its interplay with CDK11 and the mammalian target of rapamycin (mTOR) [28,48]. In addition, studies have also shown that eIF3f is downregulated in many human tumors [26,27,29], suggesting a potential role for eIF3f in regulating apoptosis and tumorigenesis.

The results of our analysis indicate that the interaction with eIF3f fosters hMSH4 protein stabilization in cells, and hMSH4 facilitates eIF3f-mediated bypass of DNA damage-induced S phase arrest, thereby ultimately promoting an early G2/M arrest. As a consequence, hMSH4-eIF3f prolongs the appearance of IR-triggered γ-H2AX foci and compromises cell survival. Consistent with these observations, eIF3f-hMSH4 is found to strongly inhibit DSB repair by the error-prone NHEJ pathway. Furthermore, our study indicates that the modulation of NHEJ-based DSB repair by hMSH4 might be channeled by the attenuation of AKT and hMRE11 responses. It is particularly pertinent that recent evidence has also hinted at the existence of a dynamic interplay between AKT and hMRE11 in NHEJ [40,41]. These studies show that DNA damage-triggered AKT activation (*i*.*e*., accumulation of pAKT-S473 at the DSB repair foci) requires hMRE11; and activated AKT upregulates hMRE11 expression, of which the outcome of this interplay is to promote NHEJ. In addition, other studies have demonstrated that hMRE11 plays an important role in NHEJ [49-51]. The DNA-dependent Protein Kinase Catalytic Subunit (DNA-PKcs) is known to mediate AKT S473 phosphorylation and activation in response to IR [52]. Thus, it will be interesting to test whether hMSH4 plays a role in manipulating the actions of DNA-PKcs, in which inactivation of DNA-PKcs would be expected to result in the reduction of AKT activation and NHEJ impairment in hMSH4 cells. Since AKT activation is often known to promote proliferation and cell survival in response to anticancer treatment, delineation of the precise mechanisms involved with hMSH4 in the process of NHEJ will be very useful for developing new anti-cancer strategies.

The results of our present study have raised another possibility that, in addition to its role in promoting meiotic HR, hMSH4 may also safeguard genome integrity by limiting the use of NHEJ in meiotic DSB repair. Expression of the hMSH4 counterpart in mouse is upregulated at the initiation of meiotic recombination and forms foci on meiotic chromosomes at the early stages of meiotic prophase I [22,53]. However, the number of Msh4 foci, most likely marking DSB repair events, on meiotic chromosomes in male mice is far greater than the numbers of crossover events. Also of note is that the majority of chromosome pairings in *Msh4*^−/−^ males are between non-homologous chromosomes [53]. As DSB repair by the homology-based repair pathway represents a critical event that precedes chromosome pairing, our current observation supports a scenario that provides a mechanism to limit the use of NHEJ (or alternative NHEJ) in meiotic DSB repair. Conversely, hMSH4 deficiency may engage NHEJ repair of DSBs located on different chromosomes, thereby facilitating non-homologous chromosome pairing. In short, our current study has illustrated that the human MutS homologue hMSH4 plays a role in the process of DNA damage response and it promotes genome stability by restricting the use of the error-prone NHEJ pathway.

## Conclusion

Our studies indicate that the hMSH4-eIF3f interaction facilitates hMSH4 stabilization, which in turn promotes genome stability through suppressing error-prone DSB repair. Consistent with this, hMSH4 sustains γ-H2AX foci and compromises cell survival in response to IR treatment.

## Methods

### Yeast two-hybrid library screening and analysis

The human hMSH4 ORF was cloned in-frame with GAL4-BD in the pAS2-1 vector, and the resulting construct was used to perform two-hybrid screening of a human ovary cDNA library in *S*. *cerevisiae* strain Y187 [20]. Yeast two-hybrid analysis was carried out according to manufacturer’s recommendations (Yeast Protocol Handbook, Clontech). cDNA sequences encoding the full-length hMSH4, eIF3f, and relevant truncation mutants were cloned into pAS2-1, pACT2, and pGBKT7 (Clontech). Positive protein interactions were ascertained by the transcription activation of *ADE2* and *HIS3* reporter genes in *S*. *cerevisiae* strain AH109 (Clontech).

### Cell culture and cell lysate

All cell lines were maintained in DMEM (Thermo Fisher Scientific, Rockford, IL) with 5% fetal bovine serum (Atlanta Biologicals, Lawrenceville, GA), 5% newborn bovine serum (Sigma, St. Louis, MO), and 1X penicillin/streptomycin (Invitrogen-Gibco, Carlsbad, CA) at 37°C with 5% CO_2_. CelLytic-M Mammalian Cell Lysis/Extraction Reagent (Sigma) supplemented with 1X protease inhibitor cocktail (Thermo Fisher Scientific) was used to make whole cell lysates. The nuclear and cytoplasmic fractionation was performed by the use of NE-PER Nuclear and Cytoplasmic Extraction Reagents Kit (Pierce). The Washington State University Institutional Review Board has approved the use of human cell lines in this study.

### Mammalian expression constructs

The coding sequence of eIF3f was cloned into pPuro-Flag [35]. Specifically, the eIF3f coding sequence was amplified by the use of primers eIF3fF1BamH (5’-CGCGGATCCATG GCCACACCGGCGGTACCAGTAAG) and eIF3fR1088EcoR (5’-CGGAATTCTGCTTGGGGTCCATTCACAGGTTTA). The full-length hMSH4 and hMSH4sv coding sequences were cloned into pMyc-CMV (Clontech). The pcDNA6(BSD)/Flag-hMSH4 expression construct was generated previously [20]. The coding sequences of hMSH4 aa1-183 and aa848-936 fragments were cloned into pPuro-FLAG [22] and pECFPC1 (Clontech), respectively. All constructs were sequence validated before use, and the expression of corresponding proteins was confirmed by Western blot analysis. RNAi-mediated eIF3f silencing was accomplished by the use of shRNA encoding constructs, pmH1P-neo/eIF3f sh-1 and pmH1P-neo/eIF3f sh-2, targeting eIF3f transcript at nucleotide positions 629–649 and 677–697, respectively.

### Cell transfection and stably transfected cell lines

All transfections were carried out by a standard calcium-phosphate procedure [54]. Transfected cells were harvested 48 hrs after transfection, unless otherwise specified. To generate a 293T/Flag-eIF3f cell line, 293T cells were transfected with pPuro-Flag/eIF3f. Stable eIF3f transfectants were selected by 2.5 μg/ml puromycin (Invitrogen) for approximately one month, single colonies were expanded and the expression of eIF3f was validated by immunoblotting. The established 293T/Flag-eIF3f stable cell line was then transfected with pcDNA6(BSD)/Flag-hMSH4 to generate a stable cell line expressing both eIF3f and hMSH4 (*i*.*e*. 293T/eIF3f-hMSH4), in which 10 μg/ml blasticidin (Invitrogen) was used for the selection. The expression of desired proteins in 293T/eIF3f-hMSH4 cells was validated with Western blot analysis.

### SDS-PAGE, Western blotting, co-immunoprecipitation (co-IP), and antibodies

Cell lysates or immunoprecipitates were resolved by SDS-PAGE, transferred onto nitrocellulose membranes (Bio-Rad Laboratories, Hercules, CA), and were subsequently used for immunoblotting. All blots were first blocked with 3% milk in 1xTBS containing 0.1% Tween-20 before addition of the primary antibodies. Immunoprecipitates were captured by incubation with 50% slurry of BSA-saturated Protein A/rProtein G-Agarose beads (Invitrogen). Antibodies used in this study include: α-Flag M2 (Sigma), α-α-tubulin (Sigma), α-GST (GE Healthcare Life Sciences, Piscataway, NJ), α-eIF3f (Rockland Immunochemical Inc.), α-Myc (Clontech), and α-hMSH4 [20], α-p-AKT (Ser473) (Cell Signaling Technology, Danvers, MA), α-pS15-p53 (Cell Signaling Technology), α-total p53 (Cell Signaling Technology), α-phospho-Chk2 Thr68 (Cell Signaling Technology). α-hMRE11 (Novus Biologicals, Littleton, CO), α-HDAC3 (Abcam), α-prefolding 3 (VBP1) (K-13, Santa Cruz), α-Rad51 (ab-1) (Novus).

### Generation of recombinant protein and GST pull-down assay

Proteins were generated according to guidelines in the BL21-CodonPlus Competent Cells Instruction Manual (Stratagene, La Jolla, CA) with minor modifications. GST pull-down assay was performed as previously described [35]. The four eIF3f deletion constructs were kindly provided by Jiaqi Shi [29]. Bacterial expression construct pET-28a/hMSH4 was created previously [20].

### Ionizing radiation (IR) exposure, cell cycle and cell survival analysis

Irradiation was carried out at room temperature with a cobalt-60 source at a dose rate of 6.6 or 4.45 Gy/min (Nuclear Radiation Center, Washington State University). Irradiated cells were harvested at indicated time points and washed twice with 1xPBS before fixed in 70% EtOH at −20°C. Cell cycle analysis was performed with the standard propidium iodide staining procedure. For each condition, 10,000 cells were analyzed by a Becton Dickinson FACSCalibur with the CellQuest Pro Software (BD, Franklin Lakes, NJ). The clonogenic survival assay was performed in triplicate 6-cm tissue culture dishes each contained 500 control or treated cells. Cells were maintained in culture for approximately 10 to 14 days to allow colony formation. Colonies with at least 50 cells were visualized and recorded. Survival fraction was determined in reference to the untreated control for each cell line.

### γ-H2AX foci analysis

Analysis of γ-H2AX foci formation was carried out as described previously [55] with α-γ-H2AX antibody (Upstate, Billerica, MA) (1:1000) and the secondary antibody Alexa Fluor 488 goat anti-mouse IgG (Invitrogen) (1:2000). Images were analyzed with a Leica Leitz DMRB fluorescence microscope (Leica Microsystem, Richmond, IL), captured by a Leica DFC310 FX camera, and processed by the Leica LAS V3.8 software (Leica Microsystem).

### In vitro and in vivo NHEJ assays

Cell extracts were prepared from one-liter suspension cultures. The *in vitro* NHEJ reaction was performed with 10 μg of cell extracts and 0.3 μg of *Sal*I-digested pMyc-CMV (Clontech) DNA in NHEJ buffer supplemented with 1 mM ATP and 1 mM DTT in a final volume of 20 μl [56]. Reactions were carried out at room temperature and terminated with the addition of 2 μl of 0.5% SDS, 2 μl of 0.5 M EDTA, and 1 μl of 10 mg/ml protease (Sigma) followed by a 30-min incubation at 37°C. NHEJ products were separated by agarose gel electrophoresis and were visualized and quantified after ethidium bromide staining.

The *in vivo* NHEJ locus was created by cloning two I-*Sce*I recognition sites as inverted repeats in between the only start codon and a linker sequence placed in front of an ATG-less GFP coding sequence in such a way that the ATG start codon is not in-frame with the GFP coding sequence. To generate a reporter cell line, this NHEJ locus was cloned into pPuro-Flag and the resulting reporter construct was stably integrated into 293T cells to create the NHEJ reporter cell line 293T/#8-1. To perform the *in vivo* NHEJ analysis, 293T/#8-1 cells were transiently transfected with 4 μg pCBA-(I-*Sce*I) plasmid DNA by the use of Amaxa Nucleofector (Lonza Group Ltd, Allendale, NJ). The appearance of GFP positive cells (relative NHEJ activity) was analyzed and recorded by FACS analysis of 25,000 or 100,000 cells (FACSCalibur, Becton Dickinson).

## Abbreviations

IR: Ionizing radiation; DSB: Double-strand break; MMR: Mismatch repair; NHEJ: Non-homologous end joining; hMSH4: Human MutS homologue 4; eIF3f: Eukaryotic translation initiation factor 3, subunit F.

## Competing interests

The authors declare that they have no competing interests.

## Authors’ contributions

YLC, XW, and YX carried out the experiments. YC and XW participated in the analysis and interpretation of experimental results as well as the preparation of the manuscript. CH conceived of the study and participated in the design and execution of experiments in addition to manuscript preparation. All authors read and approved the final manuscript.

## Authors’ information

School of Molecular Biosciences, Mail Drop 64–7520, College of Veterinary Medicine, Washington State University, Pullman, WA 99164, USA.
